# Bladder Washing Cytology in the Detection of High-Grade Prostatic Adenocarcinoma: A Case Report

**DOI:** 10.7759/cureus.98444

**Published:** 2025-12-04

**Authors:** Gabriel Pastrana, Camille L Santiago Negron, Suheidy Cardona, Juan C Santa Rosario, María D Rivera Rolón

**Affiliations:** 1 Pathology and Laboratory Medicine, University of Medicine and Health Sciences, Basseterre, KNA; 2 Pathology and Laboratory Medicine, Medical Sciences Campus, University of Puerto Rico, San Juan, USA; 3 Pathology and Laboratory Medicine, CorePlus, Carolina, USA

**Keywords:** bladder washing cytology, high-grade prostate cancer, nkx3.1 immunocytochemistry, prostatic adenocarcinoma, urine cytology

## Abstract

Bladder washing cytology is a valuable diagnostic tool primarily used in the evaluation of urothelial malignancies. However, its role in detecting metastatic or secondary malignancies, such as high-grade prostatic adenocarcinoma, is less commonly recognized. We present the case of an 82-year-old man whose post-cystoscopy bladder washing cytology revealed atypical glandular cells. Immunocytochemical staining showed strong NKX3.1 nuclear positivity, suggesting a prostatic origin. Subsequent transurethral resection of the prostate confirmed high-grade prostatic adenocarcinoma with intraductal carcinoma and a cribriform growth pattern, classified as International Society of Urological Pathology (ISUP) Grade Group 4 (Gleason score 4+4=8). Notably, NKX3.1-positive malignant cells were also identified in a follow-up voided urine sample. This case underscores the potential utility of urine cytology, particularly when supplemented by immunocytochemistry, in the diagnosis of prostate cancer.

## Introduction

Prostate cancer is the most common non-skin malignancy diagnosed in men in the United States and is a leading cause of cancer-related morbidity and mortality worldwide [1]. Early detection relies primarily on prostate-specific antigen (PSA) testing, with histopathologic confirmation obtained through transrectal or transperineal prostate biopsy [1]. While these approaches are effective in most cases, atypical presentations and diagnostic pitfalls can delay recognition, particularly in patients with coexisting urinary symptomatology or other genitourinary pathologies.

Urine cytology is a well-established, non-invasive diagnostic tool primarily intended for detecting high-grade urothelial carcinoma, especially in patients with hematuria or a history of urothelial neoplasia [2]. The Paris System for Reporting Urinary Cytology (TPS) standardizes interpretation by focusing on the identification of high-grade urothelial carcinoma and its precursors [2]. However, urine cytology may occasionally reveal malignant cells of non-urothelial origin, including secondary involvement by prostatic adenocarcinoma or other malignancies [3]. Such findings are uncommon and may be overlooked, particularly when clinical suspicion for prostate cancer is low or when urinary cytology is performed for unrelated indications. Supporting this, Tang et al. found that only 19.5% of urine cytology specimens from patients with histology-proven prostate cancer showed atypia or higher (C3+), and just 3.4% were suspicious or higher (C4+) [4]. The study also demonstrated that urine cytology is less sensitive in patients with low PSA, with a negative correlation between PSA ≤4.0 ng/mL and positive cytology findings [4]. In comparison, higher PSA levels (>20 ng/mL) were positively correlated with detection [4]. 

This report describes a case of high-grade prostatic adenocarcinoma, International Society of Urological Pathology (ISUP) Grade Group 4 [5], initially suspected by bladder washing cytology and confirmed with prostate biopsy. The case highlights the importance of considering prostatic origin when atypical glandular cells are identified in urinary cytology specimens and demonstrates the diagnostic value of immunohistochemical markers, such as NKX3.1, in establishing the site of origin.

## Case presentation

Initial evaluation

An 82-year-old Hispanic male patient underwent bladder washing cytology following cystoscopy. Cytological evaluation revealed the presence of malignant cells of non-urothelial morphology. The specimens were received in our laboratory as an external referral with limited accompanying clinical history, which is common in private practice. Immunocytochemical staining for NKX3.1 demonstrated strong nuclear positivity, raising suspicion for a prostatic primary. A subsequent PSA test showed a level of 3.180 ng/mL. Guided by the ancillary study-supported cytologic findings and the high index of suspicion of prostate cancer, the patient underwent transurethral resection of the prostate (TURP).

Cytopathologic findings

**Figure 1:**
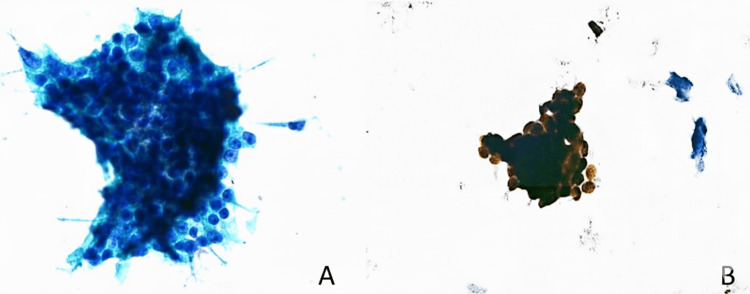
Atypical glandular cells in bladder washing preparation. (A) Liquid-based cytology preparation (Pap stain, 60x). (B) Strong nuclear positivity for NKX3.1 immunocytochemistry, highlighting the malignant cells (40x).

The bladder washing specimen (60 mL, yellow, non-bloody) was processed using liquid-based cytology. Examination revealed both solitary and clustered atypical glandular cells exhibiting a high nucleus-to-cytoplasmic ratio, irregular chromatin, and prominent nucleoli (Figure 1A). No cytologic features of high-grade urothelial cancer were observed. Given the cytomorphologic features suggestive of a possible prostatic origin, NKX3.1 immunocytochemistry was performed on a Papanicolaou-stained slide. The significant nuclear positivity for NKX3.1 in the atypical cells supported a diagnosis of prostatic cancer (Figure 1B). Continued malignant cell shedding into the urinary tract was confirmed by the discovery of atypical glandular cells with nuclear NKX3.1 positivity in a later voided urine specimen.

Histopathologic findings

**Figure 2:**
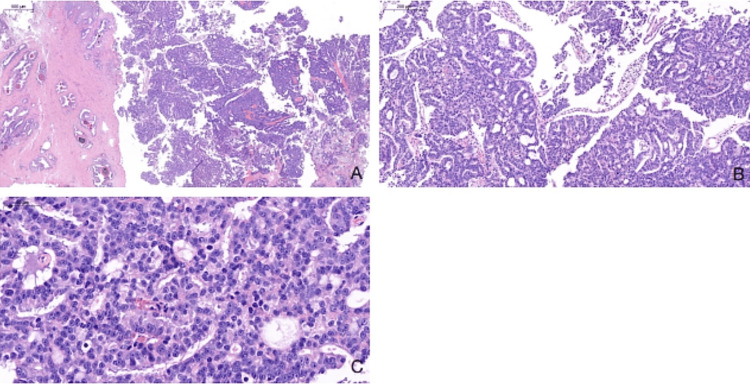
Whole slide images (WSI) showing histopathological features of prostatic adenocarcinoma in prostate tissue. (A) Low-power view showing crowded, infiltrative glands disrupting normal architecture (H&E WSI, 8.2x). (B) Cribriform pattern with fused glands and punched-out lumina, characteristic of Gleason pattern 4 (H&E WSI, 28.2x). (C) High-power image showing malignant epithelial cells with enlarged nuclei and prominent nucleoli (H&E WSI, 104.4x).

The TURP specimen, consisting of multiple tan tissue fragments measuring 2.0 × 0.8 × 0.5 cm, showed high-grade acinar adenocarcinoma of the prostate. The tumor was classified as ISUP Grade Group 4 (Gleason score 4 + 4 = 8). Histologic examination also showed the presence of both intraductal carcinoma and a cribriform growth pattern (Figures 2A-2C).

**Figure 3:**
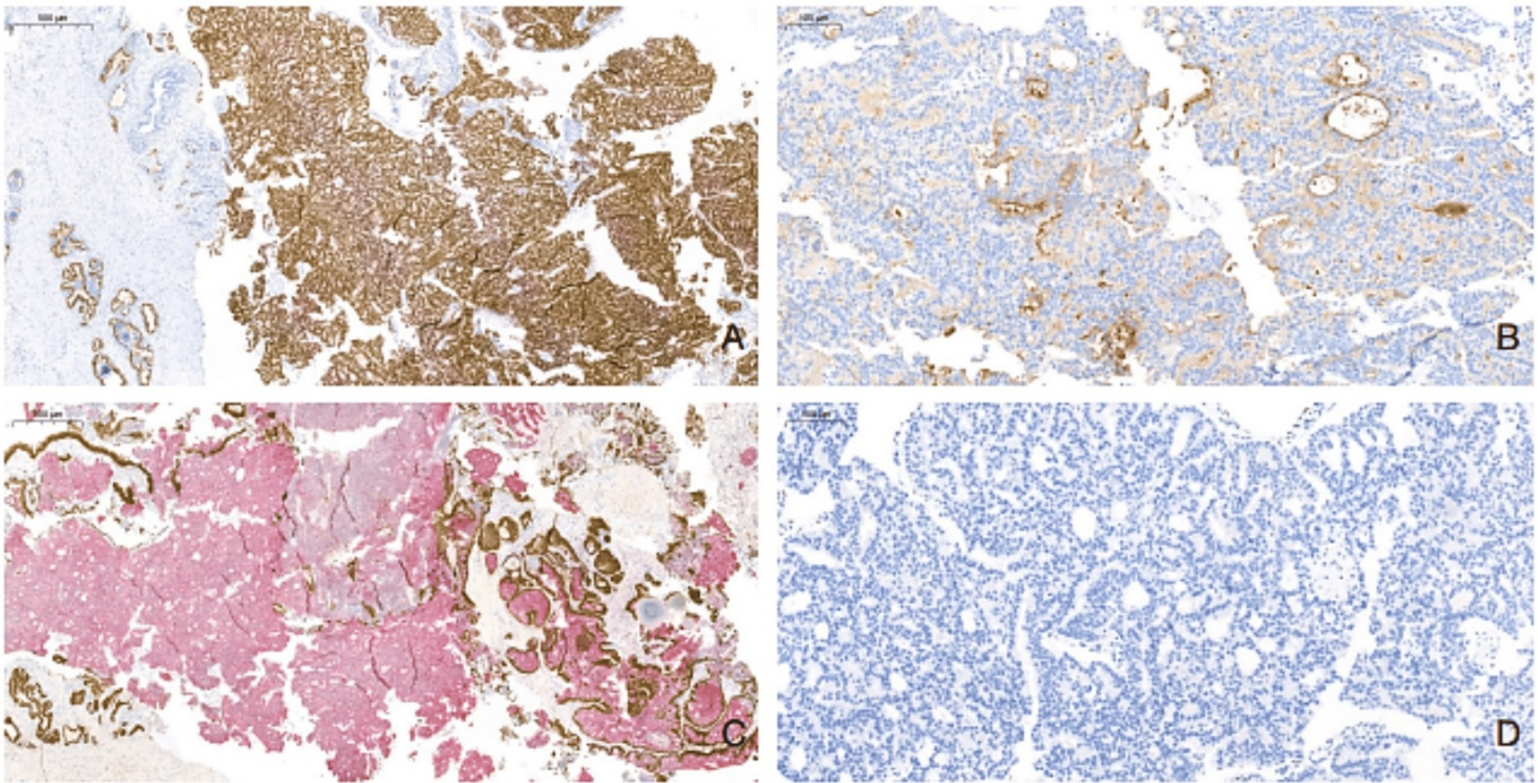
TURP biopsy immunohistochemical profile. (A) NKX3.1 diffuse nuclear positivity in tumor cells, consistent with prostatic lineage (WSI, 10.7x). (B) PSA is strongly positive in the cytoplasm. (C) PIN-4 demonstrates positive cytoplasmic P504S (WSI, 9.3x). (D) GATA-3 is negative in tumor cells (WSI, 40x). TURP, transurethral resection of the prostate; WSI, whole slide image

Immunohistochemical analysis revealed that the tumor cells were negative for 34βE12, p63, CK7, CK20, and GATA-3, and positive for NKX3.1, PSA, P504S, and cytokeratin AE1/AE3 (Figures 3A-3D). These findings further supported the diagnosis of high-grade prostatic adenocarcinoma, while effectively ruling out urothelial carcinoma.

**Figure 4:**
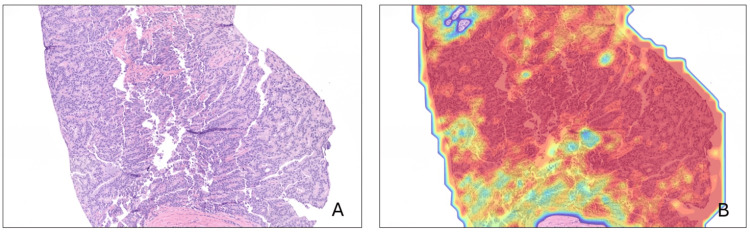
Prostate core needle biopsy tissue. (A) H&E WSI with high-grade prostatic acinar adenocarcinoma (12x). (B) AI-generated heatmap overlying the same area of prostatic acinar adenocarcinoma using the Ibex Galen platform (Ibex Medical Analytics, Tel Aviv, Israel).

The patient was later evaluated with an ultrasound-guided transrectal prostate core needle biopsy. The tissue obtained confirmed the presence of high-grade prostatic acinar adenocarcinoma in 9 out of 21 cores. Whole slide images (WSIs) were submitted for artificial intelligence (AI) analysis using the Ibex Galen Prostate platform (Ibex Medical Analytics, Tel Aviv, Israel), a validated AI-powered system for prostate cancer detection and grading in WSIs. This technology has demonstrated high diagnostic accuracy and reproducibility in both blinded studies and real-world clinical settings [6,7]. Figures 4A-4B show AI-generated heatmaps highlighting the areas of malignancy using the Ibex Galen Prostate platform.

## Discussion

Although prostatic epithelial cells can occasionally be seen in urine cytology preparations, the primary clinical role of urinary cytology is the detection of urothelial carcinoma cells [2]. When atypical glandular cells are found in urine, it is essential to consider and exclude a prostatic origin. In this case, additional urologic testing was guided by NKX3.1-supported cytology results, leading to a confirmed diagnosis of prostatic adenocarcinoma.

The expression of the NKX3.1 nuclear transcription factor is highly specific for prostate tissue and is routinely used as an immunohistochemical marker [8]. It is considered one of the most sensitive and specific immunomarkers for prostate tumors, and both histologic and cytologic preparations have demonstrated its usefulness [9,10]. Even in specimens with limited cellularity, NKX3.1 is a reliable marker demonstrating a significantly higher sensitivity than PSA and prostate-specific antigen phosphatase (PSAP) in cytology specimens for detecting prostatic adenocarcinoma [11].

Fujita et al. investigated the detection of prostate cancer cells in urine following prostatic massage using a multiplex immunofluorescence panel that included NKX3.1. According to their study, the specificity was 100%, and the sensitivity was 36% [12]. Although uncommon in routine practice, this phenomenon underscores the diagnostic value of cytology when paired with highly specific immunomarkers.

The cytologic results in our case prompted urologic referral and assessment, followed by histologic confirmation. The presence of neoplastic cells in both bladder washing and voided urine specimens may be explained by the tumor's anatomic periurethral location, which most likely contributed to malignant cell shedding into the bladder.

Moreover, this case highlights a broader clinical interest in non-invasive diagnostic techniques for cancer detection. While molecular urine-based assays like PCA3, TMPRSS2:ERG, SelectMDx, and ExoDx are gaining popularity [13], cytology remains a valuable diagnostic tool because it allows for direct visualization of cellular morphology. In selected patients, particularly those for whom biopsy may be deferred or deemed inadvisable, urine cytology can serve as a valuable adjuvant when utilized alongside immunocytochemistry, particularly NKX3.1.

## Conclusions

This case demonstrates that urine cytology, particularly in patients from whom atypical glandular cells are detected in the absence of a detailed clinical history, can assist in identifying the prostatic origin of these cells when combined with NKX3.1 immunocytochemistry. This approach may be helpful in patients who present with atypical glandular cells with morphologic features suggestive of primary prostatic origin. In these situations, urine cytology with NKX3.1 immunocytochemistry may prompt timely urologic evaluation. Further studies in larger patient cohorts are warranted to evaluate its diagnostic performance and potential role in clinical practice.
